# A Role of DNA Methylation within the CYP17A1 Gene in the Association of Genetic and Environmental Risk Factors with Stress-Related Manifestations of Schizophrenia

**DOI:** 10.3390/ijms232012629

**Published:** 2022-10-20

**Authors:** Margarita Alfimova, Nikolay Kondratyev, Galina Korovaitseva, Tatyana Lezheiko, Victoria Plakunova, Marina Gabaeva, Vera Golimbet

**Affiliations:** Mental Health Research Center, 115522 Moscow, Russia

**Keywords:** DNA methylation, schizophrenia, risk locus, stress, *CYP17A1*, *AS3MT*, executive functions

## Abstract

As genetic and environmental influences on schizophrenia might converge on DNA methylation (DNAm) within loci which are both associated with the disease and implicated in response to environmental stress, we examined whether DNAm within *CYP17A1*, a hypothalamus–pituitary–adrenal axis gene which is situated within the schizophrenia risk locus 10q24.32, would mediate genetic and environmental effects on stress-related schizophrenia symptoms. DNAm within an exonic–intronic fragment of *CYP17A1* was assessed in the blood of 66 schizophrenia patients and 63 controls using single-molecule real-time bisulfite sequencing. Additionally, the VNTR polymorphism of the *AS3MT* gene, a plausible causal variant within the 10q24.32 locus, was genotyped in extended patient and control samples (*n* = 700). The effects of local haplotype, VNTR and a polyenviromic risk score (PERS) on DNAm, episodic verbal memory, executive functions, depression, and suicidality of patients were assessed. Haplotype and PERS differentially influenced DNAm at four variably methylated sites identified within the fragment, with stochastic, additive, and allele-specific effects being found. An allele-specific DNAm at CpG-SNP rs3781286 mediated the relationship between the local haplotype and verbal fluency. Our findings do not confirm that the interrogated DNA fragment is a place where genetic and environmental risk factors converge to influence schizophrenia symptoms through DNAm.

## 1. Introduction

Schizophrenia is a multifactorial neurodevelopmental disorder in which both genetic and environmental factors contribute to the risk. By now, using genome-wide association studies (GWAS), the Psychiatric Genomics Consortium (PGC) has identified 287 disease-associated loci that, when combined into a polygenic risk score, explain about 7% of the variability in susceptibility to schizophrenia [1]. In turn, environmental risk factors, combined into a polyenviromic risk score (PERS), might explain about 20% of the variance in case-control status [2]. A key question concerns the relationship between the mechanisms by which these genetic and environmental risk factors influence the pathogenesis of schizophrenia. These relationships may be different, determining the diversity of etiological and pathogenetic architecture of the disease. First, they can have independent starting points at the level of the expression of genes that trigger the pathogenic cascade, converging at the level of brain networks (equifinality) [3]. It is also possible that environmental factors change the expression of the same genes whose expression is affected by genetic variants conferring the risk of schizophrenia (additive effects). Finally, certain risk loci might act only in response to specific environmental exposures (genotype–environment interactions).

Leveraging epigenetic marks, including DNA methylation (DNAm), may help answer the above question, as the epigenetic regulation is believed to be an important mediator of early environmental influences on gene expression [4]. Indeed, research suggests that although DNA structure generally drives DNAm at variably methylated CpG sites (VMS, sites with high interindividual variation of methylation rates within a given tissue), a combination of genetic and environmental factors best explain DNAm at most of them [5,6,7].

With this in mind, we examined how clinical features of schizophrenia were affected by DNAm within a DNA fragment, where a joint effect of genetic and environment risk factors of schizophrenia on DNAm might be expected. The fragment resides within the 10q24.32 locus, the third strongest associated locus with the disease according to PGC2 GWAS [8]. The locus contains many genes. The attention of researchers is mainly attracted to the *AS3MT* gene that encodes the human enzyme As(III) S-adenosylmethionine methyltransferase catalyzing arsenic biotransformation and is a common risk factor for several psychiatric diseases, including affective disorders and epilepsy [9,10]. We instead investigated an exonic–intronic fragment (chr10:104,594,471–104,595,888, hg19) of the *CYP17A1* gene lying 5’ to *AS3MT*, in a head-to-head configuration, because of the *CYP17A1’*s involvement in the response to stress. Specifically, the *CYP17A1’*s product takes part in the synthesis of mineralocorticoids and glucocorticoids; in line with the *CYP17A1’*s function, the *CYP17A1* polymorphism has been associated with stress-related features—cortisol response and hippocampal volume [11]. As such, the DNAm of *CYP17A1*, like that of other hypothalamus pituitary adrenal axis genes [12], might be linked to stress.

The interrogated DNA fragment was chosen because of its high regulatory activity, as evidenced by the presence of DNaseI Hypersensitivity sites (DHS) and a number of transcription factor binding sites (TFBS) (UCSC genomic browser [13]). It contains expression and splicing quantitative trait loci (QTLs) for *AS3MT* and other nearby genes in several tissues [14]. There are also multiple chromatin interactions of the fragment with surrounding DNA segments, including the *AS3MT* locus [15]. DNAm might impact on the fragment’s regulatory influence, e.g., through changing the binding of DNAm-sensitive transcription factors such as CTCF.

We expected therefore that the DNAm within this fragment would reflect additive or epistatic effects of the local schizophrenia risk genotype and early life exposures on stress-related phenotypes of the disorder. To test the hypothesis, we evaluated DNAm of each cytosine within this fragment in the peripheral blood of patients with schizophrenia and healthy subjects, using single-molecule real-time bisulfite sequencing (SMRT-BS [16]) and identified VMS. Next, in the patients, we assessed the influence of a local haplotype and polyenviromic risk score (PERS) and their interaction on DNAm at these sites. We then investigated the effects of DNAm at the VMS on stress-related phenotypes. Considering that glucocorticoids primarily affect the hippocampus and prefrontal cortex, where their receptors are most abundant, we chose cognitive phenotypes in which these brain regions play a key role, namely, deficits of declarative episodic memory (hippocampus) and executive functions (prefrontal cortex) [17]. The stress-related phenotypes such as depression and suicidal ideation and behavior (SIB) [18] were also assessed. Finally, a potential mediating role of DNAm at the VMS in the relations between genetic and environmental factors and phenotypes was examined.

As an auxiliary analysis, we evaluated associations between the stress-related phenotypes and the VNTR (variable number of tandem repeats) polymorphism located at the 5’ untranslated region of the first exon of *AS3MT* near schizophrenia-associated SNP rs7085104 [19] in extended samples of patients and controls. This was completed to further clarify the contribution of genetic variants within the 10q24.32 locus to the DNAm and phenotypes, since this locus demonstrates an unusually strong linkage disequilibrium (LD) [20] and the *AS3MT* polymorphism is associated with DNAm of a large region around the gene [21]. The rationale for interrogating the VNTR was its high potential to be a schizophrenia-causative variant within the *AS3MT* gene. Prior research [22,23] has shown that the VNTR regulates the alternative splicing of a brain-unique isoform AS3MT^d2d3^ lacking arsenite methyltransferase activity; this isoform has higher levels in individuals with schizophrenia compared to controls; it is upregulated during human stem cell differentiation toward neuronal fates; and it reduces densities of mushroom dendritic spines in cultured primary hippocampal neurons. In addition, the VNTR predicts DNAm within the *AS3MT* gene [22], influences brain activation of the prefrontal and cingulate cortex during a working memory task [24], and has shown an interaction effect with obstetric complications on negative symptoms in women with schizophrenia [25]. The similar effects of the local and VNTR genotypes on DNAm and behavior could be expected owing to the strong LD. On the other hand, if the VNTR and the local genotypes act through different molecular mechanism on the activity of the same gene (*AS3MT*), their additive effect on schizophrenia symptoms might take place. Finally, the genotypes can show different effects if they tag different causal variants and genes.

## 2. Results

### 2.1. Sample Characteristics

Demographic and clinical characteristics of the samples are presented in Table 1. The patients had lower education levels compared to controls and demonstrated significant cognitive deficits.

Such early environmental risk factors as season of birth (SOB), obstetric complications (OC), ethnicity, urbanicity, immigration, and adverse childhood experiences (ACE) were considered. Of them, SOB, OC, and ACE demonstrated variability in our samples and were chosen to calculate PERS. The analysis of pairwise relationships of the environmental risk factors and potential confounders showed that they were not completely independent, with some relationships being seen between OC and SOB (χ^2^ = 4.24, *p* = 0.039; more OC in winter SOB); education and ACE (χ^2^ = 6.11, *p* = 0.013, lower education in the presence of ACE); education and sex (χ^2^ = 5.64, *p* = 0.018; higher education in women); OC and sex (χ^2^ = 4.99, *p* = 0.021; more OC in men).

### 2.2. DNAm in Patients and Controls

**Figure 1:**
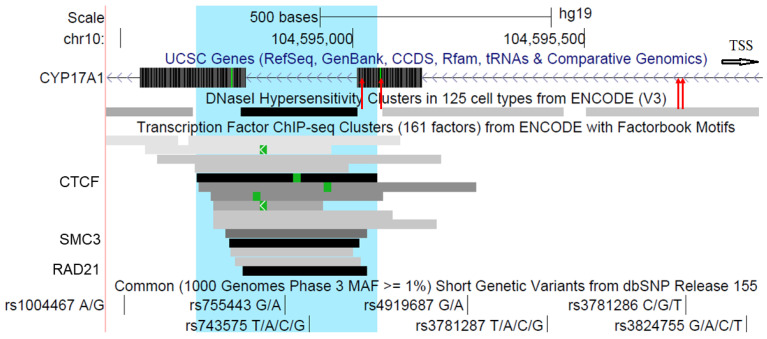
The interrogated fragment within the *CYP17A1* gene and variably methylated CpG sites (VMS) within it. The fragment view in the UCSC genomic browser ([13], http://genome.ucsc.edu, accessed on 5 September 2022) is presented. Exons 2 and 3 (striped rectangles), intron 2, and parts of introns 1 and 3 (the line with arrows indicating the direction of transcription) of the *CYP17A1* gene are given; the white arrow indicates where the transcription start site (TSS, chr10:104,597,290) is located. VMS positions are shown with red arrows. TFBS are shown as grey and black rectangles. The CTCF binding site is highlighted (blue).

The DNAm of 401 cytosines was determined. All CpH were hypomethylated. Among CpG, four were variably methylated (0.20 ≤ DNAm ≤ 0.80) (Figure 1, Table 2 and Table S1 (Supplementary Materials)). Of them, two cytosines with coordinates chr10: 104,595,032 and chr10:104,595,063 (hereinafter CpG_5032 and CpG_5063) were located at the end of the TFBS and DHS cluster (chr10:104,594,761–104,595,010), between unmethylated and hypermethylated DNA regions. Specifically, CpG_5032 was situated at the end of the RAD21 binding site (chr10:104,594,764–104,595,031), while CpG_5063 was located at the end of the CTCF binding site (chr10:104,594,666–104,595,053). The other two VMS were CpG-SNP rs3781286 (CpG-SNP_5719, C > T) and an adjacent CpG at chr10:104595714 (CpG_5714).

DNAm at the VMS did not correlate with each other, except for CpG_5714 and CpG-SNP_5719 (Spearman correlations conditioned on diagnosis: rho = 0.57; *p* < 0.001). Stepwise logistic regression, which included DNAm at the four VMS as predictors of diagnosis, with age, sex, and coverage as covariates, showed that the patients significantly differed from healthy controls by an increase in DNAm at CpG_5714 (β = 2.91; SE = 1.10; 95% bias corrected accelerated confidence intervals [bca 95% CI] 0.75–4.96; *p* = 0.009). Patients also tended to show lower DNAm at CpG-SNP_5719 than controls (β = −0.81; SE = 0.41; [bca 95% CI] −1.58–0.009; *p* = 0.054) (see Table S2 for the regression model and multicollinearity testing).

### 2.3. Genetic and Environmental Influences on DNAm at VMS

The TGT haplotype (rs743575-rs4919687-rs3781287) was identified as a local schizophrenia risk haplotype based on the linkage in European populations of these SNPs with rs11191419, a tagging SNP for this schizophrenia risk locus [8], using the DLINK tool [26]. The other common haplotypes were GAG and TGG. Information on SNPs within the interrogated fragment can be found in Table S3.

According to the Kolmogorov–Smirnov criterion, DNAm at CpG_5032 and CpG_5063 were normally distributed, while DNAm at CpG_5714 demonstrated asymmetry and was brought to a normal distribution by cubing. DNAm at these three sites was analyzed by linear regression, in which haplotype, PERS, and the interaction of haplotype (presence/absence of the risk allele TGT) with PERS were predictors. For regression coefficients, bca 95% CI and bca *p*-values were calculated based on 1000 bootstrap samples. To select confounders, we assessed the influence of sex, age, education, smoking, history of substance use, and coverage on DNAm. Education and coverage turned out to be significantly associated with DNAm at CpG_5063 and CpG_5719, respectively. Education was therefore included as a covariate into the model for CpG_5063. DNAm at CpG-SNP_5719 had an almost bimodal distribution. For this reason, the effect of haplotype on DNAm at CpG-SNP_5719 was determined by the Kruskal–Wallis test and post hoc Dunn’s test; the effect of PERS was assessed using Spearman correlations conditioned on the risk allele and coverage. Bonferroni correction for a family of four VMS sites was applied to all *p*-values of interest.

**Figure 2:**
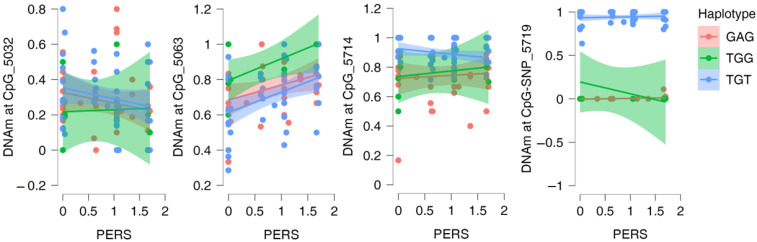
Relations of DNA methylation (DNAm) at variably methylated sites with local haplotypes (rs743575-rs4919687-rs3781287) and polyenviromic risk scores (PERS). CpG names contain last digits of their coordinates on chromosome 10, hg 19. For each haplotype, a regression line with 95% CI (shaded areas of the same color) are shown.

All models are presented in Figure 2 and Table S4. Neither local haplotype nor PERS affected DNAm at CpG_3032. An additive effect of haplotype and PERS on DNAm at CpG_5063 was found. DNAm of TGG alleles was significantly higher than that of TGT alleles (β = 0.20; SE = 0.07; [bca 95% CI] 0.07–0.34; p_bonf_ < 0.004). DNAm here also increased with increasing PERS values (β = 0.09; SE = 0.04; [bca 95% CI] 0.02–0.16; p_bonf_ = 0.040). When each of the three risk factors was analyzed instead of PERS, the additive effect of haplotype and environment was confirmed for ACE (ANOVA; haplotype: F = 5.000, df = (2, 78), *p* = 0.009, η^2^_p_ = 0.114; ACE: F = 9.513, df = (1, 78), *p* = 0.003, η^2^_p_ = 0.109). DNAm was higher if ACE was present. SOB and OC were not associated with DNAm at CpG_5063.

An allele-specific DNAm was found at CpG_5714 (GAG vs. TGT: β = −0.35, SE = 0.09, [bca 95% CI] −0.53–−0.19; p_bonf_ < 0.004) and CpG-SNP_5719 (Kruskal–Wallis Test: H = 68.50, p_bonf_ < 0.004; TGG vs. TGT, Dunn’s test: z = 4.40, p_bonf_ < 0.001; GAG vs. TGT, Dunn’s test: z = 7.94, p_bonf_ < 0.001). DNAm was higher in the presence of the schizophrenia risk allele TGT compared with the other two alleles in accord with the linkage between the TGT haplotype and the allele C of rs3781286 creating CpG at chr10: 104595719 (D′ = 1.0; R^2^ = 0.92; χ^2^ = 929.98; *p* < 0.0001 [26]).

In the controls, we could only check the influence of the haplotype. Its effect (GAG versus TGT) was significant or approached the threshold of significance for all four VMS, the TGT risk allele showing higher DNAm (Table S5).

### 2.4. DNAm and Stress-Related Phenotypes

A few patients had a depression score (PANSS G6) ≥ 4 (Table 1), so depression was not analyzed further. A preliminary analysis showed that sex, age, and education were significantly associated with the other six phenotypes—verbal fluency, episodic verbal memory, cognitive flexibility, cognitive inhibition, and SIB. We therefore included these characteristics in the regression models as covariates. We used linear and logistic regression to assess the effects of DNAm on the cognitive variables and SIB, respectively. For these analyses, DNAm was averaged across two alleles in case of heterozygosity. Stepwise regressions were performed with DNAm at each of the four VMS, sex, and age as predictors. Education was also included into the models of the cognitive measures. Bca 95% CI and bca *p*-values were calculated based on 1000 bootstrap samples.

**Figure 3:**
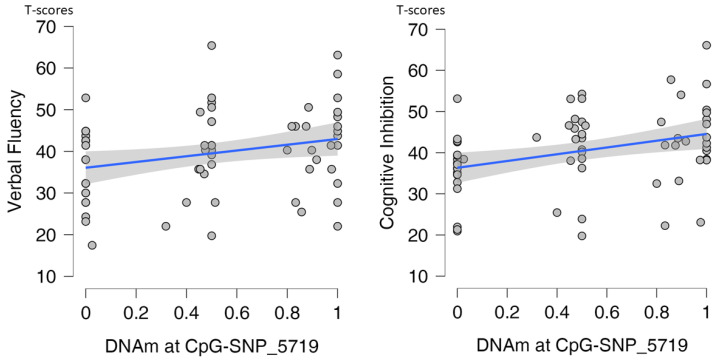
Relations of DNA methylation (DNAm) at CpG-SNP_5719 with cognitive phenotypes in patients. Regression lines (blue) with 95% CI (grey shaded areas) are shown.

**Figure 4:**
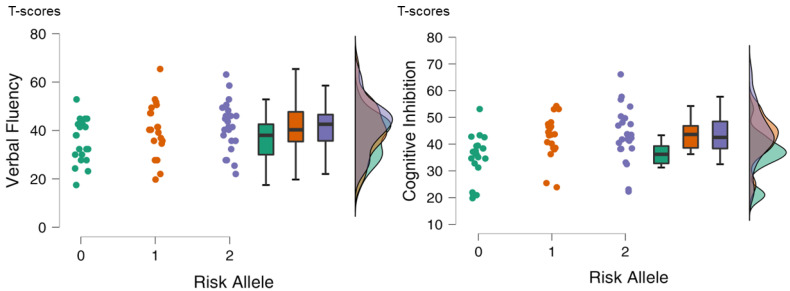
Relations of risk genotype defined as the number of the risk allele TGT (Risk Allele) with cognitive phenotypes in patients. For each genotype, the scatterplot, boxplot (mean, SD), and density are shown.

In patients, after a Bonferroni correction for a family of six phenotypes, DNAm at CpG-SNP_5719 was associated with verbal fluency (β = 7.69; SE = 2.47; [bca 95% CI] 2.68–12.11; p_bonf_ = 0.036) and cognitive inhibition (β = 8.14; SE = 2.74; [bca 95% CI] 2.99–13.63; p_bonf_ = 0.024). Higher DNAm was associated with better cognitive performance (Figure 3). When the local genotype, PERS, and genotype X PERS interaction term were included into the models instead of DNAm (genotype was coded as the number of alleles with the risk haplotype TGT—0, 1, 2), the genotype predicted the same two phenotypes: verbal fluency (β = 3.04; SE = 1.11; [bca 95% CI] 0.91–5.32; p_bonf_ = 0.018) and cognitive inhibition (β = 3.94; SE = 1.35; [bca 95% CI] 1.70–7.16; p_bonf_ < 0.004). The risk genotype was associated with higher cognitive scores (Figure 4). For the full models, see Tables S6 and S7. Of note, the risk genotype did not influence the severity of the PANSS positive, negative, or general psychopathological symptoms (Table S8).

We, therefore, ran separate mediation analyses for verbal fluency and cognitive inhibition, with DNAm at CpG-SNP_5719 as a mediator of the local genotype’s effects on cognitive functions and age, sex, and education as background confounders (for the full models, see Tables S9 and S10, Figures S1 and S2). The results supported the indirect effect of the risk genotype on verbal fluency through DNAm (direct effect: β = −0.98, SE = 0.51, [bc 95% CI] −3.06–−0.20, *p* = 0.093; indirect effect: β = 1.08, SE = 0.50, [bc 95% CI] 0.36–3.34, *p* = 0.031; total effect: β = 0.22, SE = 0.11, [bc 95% CI] 0.02–0.42, *p* = 0.036) but not on cognitive inhibition. In similar analyses in controls, neither DNAm nor risk genotype influenced cognition (Tables S11 and S12).

### 2.5. AS3MT VNTR and Stress-Related Phenotypes

Frequencies of VNTR genotypes in the patient and control groups were in accordance with the Hardy–Weinberg law; the minor allele (V2) frequencies were 0.41 and 0.44 in patients and controls, respectively. There was no significant difference between groups in genotype frequencies (χ^2^ = 1.29; df = 2; *p* = 0.525).

We first assessed the influence of VNTR on DNAm in patients and controls (stepwise regression with genotype and diagnosis as factors). The VNTR did affect DNAm at CpG_5714 (V2V2 vs. V3V3: β = −0.29, SE = 0.08, [bca 95% CI] −0.43–−0.11, p_bonf_ = 0.020) and CpG-SNP_5719 (V2V2 vs. V3V3: β = −0.84, SE = 0.07, [bca 95% CI] −0.92–−0.61, p_bonf_ = 0.004; V2V3 vs. V3V3: β = −0.43, SE = 0.06, [bca 95% CI] −0.52–−0.29, p_bonf_ < 0.004). However, there were neither any VNTR main effects on the stress-related phenotypes in the DNAm or extended groups of patients and controls, nor did we find any interaction effects of VNTR and PERS on the phenotypes in the DNAm or VNTR groups of patients (results of regression analyses are not shown as only demographic variables were retained in the best models).

## 3. Discussion

Given the location of interrogated fragment in the schizophrenia risk locus and simultaneously within the *CYP17A1* gene involved in the response to stress, we expected that local haplotypes and early environmental risk factors would have an additive or interaction effect on DNAm within the fragment and, through the DNAm, on stress-dependent phenotypes of schizophrenia. The hypothesis was not confirmed. We did find a variably methylated site (CpG_5063), where the variation in DNAm was explained by an additive effect of the local haplotype and environmental risk, specifically ACE; however, DNAm at this site was not associated with schizophrenia or its stress-related phenotypes. It is, however, noteworthy that CpG_5063 is located within the gene body, at the end of the DHS; as such, its DNAm might be related to a stress-dependent activity of the fragment.

We also found that the local genotype and allele-specific methylation at CpG-SNP_5719 was associated with executive functions, namely initiation (verbal fluency) and cognitive inhibition, in patients but not healthy controls. A conventional mediation analysis confirmed the DNAm-mediated indirect effect of genotype on verbal fluency but not on cognitive inhibition. This might be due to the known component nature of cognitive inhibition and the fact that individual inhibitory control processes differentially impact on lexical–semantic retrieval underlying semantic verbal fluency [27].

Thus, DNAm seems to be not only a marker but also a mediator of the genotype’s influence on semantic verbal fluency. Interestingly, although DNAm at the CpG-SNP_5719 was associated with both the local risk allele within the *CYP17A1* gene and with the VNTR within the *AS3MT* gene, the cognitive performance depended only on the local genotype. It is unexpected in light of the strong LD in the region and the fact that the local SNPs and rs7085104 in close proximity to the VNTR represent expression and methylation QTL for the same genes in the frontal cortex (*AS3MT, BORCS7*) [14] and for the same CpG sites in the blood [28]), including cg23322172 located within the intelligence GWAS hit *TMEM180* [29]. Based on our results, one might speculate that the local and VNTR genotypes tag, in addition, different causal variants and genes. In this connection, genes within the 10q24.32 locus other than *AS3MT* are of interest, specifically those located to the left of the CTCF binding site, as they might belong to another chromatin loop.

Of note, the direction of the association between the local genotype and cognition was the opposite to that which could be expected. Cognitive performance was better in the carriers of the risk allele; however, this is not uncommon in schizophrenia research. For example, an association of a risk allele with better cognitive performance has been observed for another schizophrenia risk gene—*ZNF804A* [30]. The authors suggested that *ZNF804A* was associated with a psychosis subtype in which cognition was relatively less impaired. The hypothesis was partly based on the fact that *ZNF804A* was shown to be a risk factor not only for schizophrenia but also for bipolar disorder and other psychoses, where cognitive deficits are milder than those in schizophrenia. This is similar to the case with the *AS3MT* locus, which has shown genome-wide associations with several psychiatric disorders, including affective ones [9].

Remarkably, DNAm at the four VMSs within the fragment did not highly intercorrelate and had different relationships with the local haplotypes and PERS, with seemingly stochastic (CpG_5032), genotype- and environment-dependent (CpG_5063), and allele-specific (CpG_5714 and CpG-SNP_5719) effects on DNAm being seen. Thus, our data indicate that, despite the strong LD, different factors determining DNAm variation may operate within this small DNA fragment. In addition, an increase in methylation at CpG_5714 and a downward trend at CpG-SNP_5719 found in schizophrenia patients compared to controls may be a mark of a schizophrenia-related alteration of DNAm within the fragment. These findings are of interest, given that the fragment has not been covered by the microarrays used in most research of methylome in schizophrenia patients. However, this differential methylation should be validated in independent samples.

The main limitations of our study include a relatively small size of the DNA sample, which was due to the high cost of the sequencing method used, lack of information on the environmental risk factors in controls, and exploring methylation in blood. With regard to tissue-specific methylation, it should be noted that one approach to solve the problem is to use available resources to compare DNAm at CpG of interest in the blood and brain. We had no such an opportunity because the interrogated DNA fragment was not covered by most microarrays. However, at least the DNAm levels at CpG-SNPs are thought to be highly concordant across tissues [31]. In line with this view, recent research on meQTL using the whole-genome sequencing has confirmed that rs743575, rs4919687, and rs3781287 forming the local haplotype in our study influence DNAm at CpG_5714 and CpG-SNP_5719 in the dorsal prefrontal cortex [5]. Continuing this line of reasoning, we can also consider the relationship between the DNAm at CpG-SNP_5719 (rs3781286) and the behavioral phenotypes in terms of gene expression, since rs3781286 determines both DNAm at this site and the expression of nearby genes. Specifically, our analysis of the GTEx Consortium data [14] showed that the CC genotype at rs3781286 creating CpG sites for DNAm was associated with an increase in the expression of the *AS3MT* and *BORCS7* genes in the frontal cortex (for full data extracted from GTEx, see Figure S3).

In summary, our results do not confirm that the interrogated DNA fragment might be a place where genetic and environmental risk factors converge to determine stress-related manifestations of schizophrenia through effects on DNAm. Nevertheless, the following findings could be of importance for future methylation and schizophrenia research. Regarding methylation, our detailed investigation of a small DNA fragment showed that environmental exposures and local haplotypes might differentially influence VMS situated within a short distance despite the very strong LD in the region, presumably owing to the presence of a boundary of DHS or a chromatin loop. Regarding schizophrenia research, we did not find associations of the *AS3MT* VNTR with cognitive deficits in patients, which is in line with the *AS3MT* involvement in a spectrum of psychiatric disorders with milder cognitive dysfunctions. However, other SNPs within the schizophrenia risk locus 10q24.32, specifically rs3781287 or nearby rs3781286, appeared to influence patients’ cognitive functions which deserves further research.

## 4. Materials and Methods

### 4.1. Participants

The sample where DNAm was examined (the DNAm sample) comprised 66 patients recruited at two psychiatric hospitals in Moscow. To be included, the patient had to have a diagnosis of a schizophrenia spectrum disorder according to the International Classification of Diseases (ICD-10); to be 18–45 years old and of European ancestry; and had to have no medical conditions associated with intellectual disability. DNAm data were also obtained for 63 healthy controls without a personal or family history of psychiatric disorders who fulfilled the above inclusion criteria, except for diagnosis. The extended sample genotyped for VNTR (VNTR sample) consisted of 304 patients and 466 healthy controls. The VNTR samples included the DNAm samples. All of the participants donated venous blood for DNA extraction and completed a neuropsychological battery for assessment of episodic memory and executive functions. Data on environmental risk factors, depression, and SIB were obtained only for patients. The extended sample overlapped with the sample from our previous study of the VNTR [25]. Demographic and clinical characteristics of participants are presented in Table 1. More information about the inclusion procedure can be found in [32].

### 4.2. DNAm Analysis

The DNAm analysis has been described in detail earlier [33,34]. In brief, genomic DNA was extracted and bisulfite converted using the DNeasy Blood and Tissue Kit (Qiagen, USA) and the EpiGentek Methylamp DNA Modification Kit (Epigentek Group Inc., Farmingdale, NY, USA), respectively. The sequences of primers designed with the primer3 software were as follows:

Forward—GCAGTCGAACATGTAGCTGACTCAGGTCACAAAAGGAAGGAAGATTGGGGATAATG;

Reverse—GCAGTCGAACATGTAGCTGACTCAGGTCACAAACCAATCAACCAAATCTACTAACCA.

Polymerase-chain reaction (PCR) with bisulfite-converted DNA was performed by means of an own modification of the SMRT-BS method. The CCS library preparation and sequencing were performed in the University of Washington PacBio Sequencing Services using the PacBio RSII sequencer (P6/C4 chemistry, CCS reads). After the standard post-sequencing data preparation and quality control, reads with a quality score of Q30 or more were analyzed. The alignment of the reads to the reference human genome (hg19) and the determination of methylation rates at CpGs and CpHs were made using the bismark and bowtie2 software. Haplotypes formed by local common polymorphisms with minor allele frequencies > 0.2—rs743575 (104594906), rs4919687 (104595248), and rs3781287 (104595420)—were called from sequencing data. For every participant, methylation levels of individual cytosines on each allele represented the ratio of reads with unconverted cytosine to the total amount of reads for a given cytosine. Samples with at least the 5× read depth per haplotype were analyzed. According to the literature [35], VMS were defined as sites with averaged methylation levels between 0.20 and 0.80.

### 4.3. AS3MT VNTR Genotyping

Genomic DNA from peripheral blood leukocytes was extracted by the standard phenol-chloroform method. Genotyping was performed using real-time PCR by C1000 Touch system (Bio-Rad, Hercules, CA, USA), with primers and reaction conditions described earlier [25]. The PCR fragments with two (419 bp, allele V2) and three (455 bp, allele V3) repeats were separated in an 8% PAAG.

### 4.4. Assessment of Environmental Risk Factors

To collect information about early environmental stressors, medical records were analyzed using a specially designed form. In addition, an experimenter who administered the neuropsychological battery asked patients a short, predefined question series. PERS were calculated using information on SOB, OC, and ACE. Regarding SOB, the patients were divided into individuals born in winter (December–February) versus those born in any other season. OC included preterm birth (<37 weeks), cesarean section, asphyxia/hypoxia, low birth weight (<2500 g), congenital malformations, cord complications, forceps or vacuum delivery, incubator or resuscitation, non-vertex presentation, birth injury, Rhesus incompatibility, and twin birth. According to the ACE literature [36], ACE were defined as childhood maltreatment (physical or sexual abuse, peer victimization, foster care) or family dysfunction (parental mental illness, parental substance use disorder, parental suicidality or incarceration, and domestic violence). PERS were calculated as the sum of these three risk factors, taking into account the strength of their associations with the disease. Natural logarithms of the odds ratios (OR) from a recent umbrella review [37] were used as indicators of the strength: OR for SOB = 1.04; OR for OC = 1.84; OR for ACE = 2.87.

### 4.5. Assessment of Stress-Related Phenotypes

All participants completed neuropsychological tests described earlier [34]. Here, we analyzed the following cognitive variables: the Immediate memory score, which was the sum of words recalled across five learning trials, from the Rey Auditory Verbal Learning Test (episodic verbal memory); semantic verbal fluency—the sum of animals and fruits generated within a minute each (executive functions: initiation); the time to complete the Trail Making Test, part B, TMT-B, (executive functions: cognitive flexibility); the Golden’s Interference score from the Stroop Color and Word Test [38] (executive functions: cognitive inhibition); and the number of correct answers in counting down by 2 and 5 from 200 (executive functions: working memory). Symptoms were evaluated with the Positive and Negative Syndrome Scale (PANSS, [39]). The presence of depression was defined as PANSS G6 Depression score ≥ 4 (i.e., moderate to extreme depression). Information on SIB was extracted from medical records and was entered into the same form that was used for registering environmental risk factors.

### 4.6. Data Analysis

Statistical analysis was performed with JASP 0.16 [40]. Statistical procedures used for each type of analysis are described in the respective sections of the Results.

## Figures and Tables

**Table 1:** Samples’ demographic and clinical characteristics.

Variable	DNAm Sample		VNTR Sample	
	Patients	Controls	Patients	Controls
*n*	66	63	304	466
Sex (% women)	50	52	50	59 *
Age (years)	27.49 (6.76)	26.77 (6.42)	28.76 (8.80)	28.45 (8.94)
Age range	18–45	18–45	16–63	17–60
Smokers (%)	30	52 *	-	-
Education (% tertiary)	71	89 *	63	83 **
WM (T-scores)	39.16 (11.91)	50.39 (9.50) **	38.21 (11.91)	49.90 (11.82) **
VF (T-scores)	39.46 (10.21)	50.17 (9.02) **	38.06 (10.91)	50.07 (10.21) **
EVM (T-scores)	37.03 (12.61)	50.64 (9.34) **	35.22 (12.30)	47.86 (11.19) **
Cognitive flexibility (T-scores)	37.55 (11.00)	49.99 (9.13) **	35.55 (11.73)	49.41 (9.53) **
Cognitive Inhibition (T-scores)	40.45(9.55)	50.14 (9.73) **	39.82 (10.51)	47.71 (11.09) **
Diagnosis (% F20)	82	-	80	-
Illness duration	5.95 (6.23)		6.19 (6.89)	-
PANSS P	27.38 (8.66)		25.85(8.94)	-
PANSS N	20.36 (5.89)		19.99 (7.40)	-
PANSS G	31.52 (12.06)		30.36 (12.57)	-
PANSS G6 ≥ 4 (*n*)	3	-	12	-
SIB (%)	22	-	26	-
SOB (% winter birth)	23	-	25	-
OC (%)	36	-	35	-
ACE (%)	55	-	46	-
PERS	0.81 (0.63)	-	0.71 (0.59)	-

Note. For continuous variables, mean and SD are shown. Significant differences between patient and control groups: * *p* < 0.05, ** *p* < 0.01. Abbreviations: WM—working memory; VF—verbal fluency; EVM—episodic verbal memory; PANSS—the Positive and Negative Syndrome Scale; SIB—suicidal ideation and behavior; SOB—season of birth; OC—obstetric complications; ACE—adverse childhood experiences.

**Table 2:** DNAm (mean, SD) at variably methylated sites in patients and controls.

Group	CpG_5032	CpG_5063	CpG_5714	CpG-SNP_5719
Patients	0.28 (0.19)	0.74 (0.17)	0.82 (0.16)	0.49 (0.48)
Controls	0.25 (0.18)	0.76 (0.16)	0.76 (0.21)	0.53 (0.48)

## Data Availability

The data are presented within the article and Supplementary Materials.

## Supplementary Materials

The following supporting information can be downloaded at: https://www.mdpi.com/article/10.3390/ijms232012629/s1.
